# How Risk Perceptions, Not Evidence, Have Driven Harmful Policies on COVID-19

**DOI:** 10.1017/err.2020.37

**Published:** 2020-04-20

**Authors:** Sweta CHAKRABORTY

**Affiliations:** * Principal, Adapt to Thrive, and Member, EcoHealth Alliance’s Young Professionals Council; email: sweta@adapttothrive.com.

## Abstract

COVID-19 hits all of the cognitive triggers for how the lay public misjudges risk. Robust findings from the field of risk perception have identified unique characteristics of a risk that allow for greater attribution of frequency and probability than is likely to be aligned with the base-rate statistics of the risk. COVID-19 embodies these features. It is unfamiliar, invisible, dreaded, potentially endemic, involuntary, disproportionately impacts vulnerable populations such as the elderly and has the potential for widespread catastrophe. When risks with such characteristics emerge, it is imperative for there to be trust between those in governance and communication and the lay public in order to quell public fears. This is not the environment in which COVID-19 has emerged, potentially resulting in even greater perceptions of risk.

COVID-19 hits all of the cognitive triggers for how the lay public misjudges risk. Robust findings from the field of risk perception have identified unique characteristics of a risk that allow for greater attribution of frequency and probability than is likely to be aligned with the base-rate statistics of the risk.^1^ ^,^ ^2^ COVID-19 embodies these features. It is unfamiliar, invisible, dreaded, potentially endemic, involuntary, disproportionately impacts vulnerable populations such as the elderly and has the potential for widespread catastrophe.^3^ ^,^ ^4^ When risks with such characteristics emerge, it is imperative for there to be trust between those in governance and communication and the lay public in order to quell public fears. This is not the environment in which COVID-19 has emerged, potentially resulting in even greater perceptions of risk.^5^ ^,^ ^6^ ^,^ ^7^

While research on the value-based factors, such as trust, that contribute to risk perceptions has provided great insights into how the public views and assesses risks,^8^ ^,^ ^9^ studies of public perceptions of emerging infectious diseases are still limited.^10^ What we do know is that emerging and persistent infectious diseases receive significant media attention, especially compared to other disease states that are known (eg cardiovascular disease, cancer or Alzheimer’s disease).^11^ This was true of H1NI,^12^ ^,^ ^13^ and has anecdotally so far proven true of COVID-19.

Risks are amplified or attenuated through the media through *social amplification stations*, which can range from individuals to the news media. Amplification happens in two stages: in the initial transfer of information about the risk; and in the response mechanisms in society.^14^ It is through these amplification stations that public perceptions of risks are shaped.^15^ ^,^ ^16^ These amplifications are exceptionally poignant in cases where first-hand knowledge is not tenable, such as with COVID-19, and the public is therefore reliant on the media to help ascertain the risk.^17^ ^,^ ^18^ Research shows that media coverage of a public health risk such as COVID-19 can introduce particular risk characteristics that influence public perceptions and therefore become a factor in itself in how the risk is viewed.^19^ ^,^ ^20^

In addition to the extent of media coverage is the way a public health risk is framed in the media. As mentioned above, a new, unfamiliar disease will be prescribed far higher dread than a more familiar disease (eg Lou-Gehrig’s disease), even if the more familiar disease is actually deadlier.^21^ H1N1 was also referred to in South Korean media as *shin-jong* or *new* flu.^22^ Before COVID-19 was named, it was widely referred to in the media as the *novel* coronavirus. This media attention to a “new” or “novel” infectious disease frames diseases as unfamiliar and potentially catastrophic, which trigger cognitive over-attributions of frequency and probability. This along with the social amplification of risk amplifies risk perceptions and can result in the inaccurate overemphasis of primary public health impacts.

Given heightened public awareness of the primary public health impacts associated with the novel coronavirus, media coverage has acted as a feedback loop – reinforcing the generated public awareness of these impacts. Mass media have showcased epidemiological, medical and public health perspectives on the impacts of COVID-19 – primarily the lives lost – at a serious detriment to understanding the big picture. Observationally, there has been rare inclusion of risk or behavioural science expertise in the media. Analysis of mass global media and social media coverage in the coming months and years will surely verify this observation. Even being several months into the COVID-19 outbreak, a comprehensive cost–benefit analysis of the various policies and combinations of policies put in place around the world has yet to be produced. Policies have been based on historical data, models and disproportionate emphasis on mitigating against primary public health impacts. Practitioners in risk analysis know all too well the dangers of risk analysis and policy-making in silos, and yet there has been no mainstream thorough cost–benefit analysis on COVID-19 in the context of a complex, global interconnected risk landscape.

The global risk analysis community collectively holds a plethora of knowledge and data, as well as knowledge of where data are lacking, on the primary risks related to infectious disease (eg deaths caused by the disease), as well as secondary and tertiary impacts (eg mental health impacts, lost productivity). Yet, the risk and behavioural science community has hardly been included in real-time analysis of COVID-19 and its impacts.

Policies designed after the emergence of an outbreak carry inherent risk stemming from analysis of data that are fluid and rapidly evolving. These risks can and should be minimised by ensuring that policies across various outcome scenarios are well thought out and ready for implementation long before a crisis hits. The need for proactive preparedness for an inevitable infectious disease outbreak has been consistently maintained by the infectious disease community. This lack of preparedness has resulted in disjointed policies reacting to public perceptions of risk. Specifically, a proactive risk communication plan ahead of an outbreak would have allowed for clear, consistent communication that would have quelled public fears and presumably have allowed evidence-based containment and mitigation policies to take hold.^23^ Because of a variety of factors (eg resource restrictions, varying country priorities, general complacency when there is not an outbreak), not only are evidence-based policies not dictating nation-state responses within and beyond political borders, they are rather being replaced with fear-based measures.

Consistent, clear and credible messaging helps to quell public fears. Fischhoff et al found in a survey of the US public’s understanding of Ebola following the 2014 outbreak in West Africa that the public is less likely to horribly misjudge risk when information is effectively and accurately communicated. People also have clear preferences about how they like to receive information and what sources are viewed as trustworthy.^24^

While risk tolerance varies across cultures around the globe, the public generally demands governments ensure low exposure to risks, especially if they are new or unfamiliar. Knowledge of this expectation is why proactive preparedness for anticipated risks is so critical. It has become painfully evident that this has not been the case for COVID-19. The disjointed communication response following the outbreak has most definitely perpetuated distrust in the USA and around the world. What the risk perception and communication community has urged since the development of the US Centers for Disease Control and Prevention (CDC) Crisis Communication Lifecycle^25^ – honest, accurate information (ideally researched and tested) from trusted spokespeople – has clearly been ignored at any meaningful level.

The consequences of such poor preparedness and policies have real-world implications. Governance decisions made in reaction to public fears err on quelling short-term hysteria at the expense of worse overall outcomes. The secondary and tertiary impacts stemming from COVID-19 will go well beyond the primary public health impacts. Reactive policies such as prolonged quarantines and isolations may very well increase the odds of negative outcomes. For example, Brooks et al found negative psychological effects of severe social distancing measures, including post-traumatic stress symptoms, confusion and anger. They recommended for policy-makers to minimise such measures and to communicate consistently throughout in order to reduce harm.^26^

The ripple effects of the policies put in place to mitigate against the primary public health impacts of COVID-19 may very well produce a worse overall outcomes picture. The role of the media in contributing to public perceptions of heightened risk and the reaction of policy-makers to govern based on public fears and not base-rate statistics of the disease (however fluid) will present several research opportunities in the future across multiple disciplines. This need not have been the case. It is evident that existing risk communication research has not been consistently consulted in managing the COVID-19 outbreak, nor has a comprehensive risk–benefit analysis been conducted to prevent worse overall outcomes. These measures might have offset the power of the media in shaping risk perceptions, which might have in turn resulted in preventing potentially harmful policies and misallocation of precious resources in battling this global disaster. Hopefully, the takeaways from COVID-19 will prove helpful for the next inevitable disease outbreak.

## Footnotes

1

P Slovic , “Perception of Risk” (1987) 236 Science 280 356350710.1126/science.3563507.

2

P Slovic , “Trust, Emotion, Sex, Politics, and Science: Surveying the Risk-Assessment Battlefield” (1999) 19 Risk Analysis 689 1076543110.1023/a:1007041821623.

3 Slovic, supra, note 1.

4 Slovic, supra, note 2.

5

R Lofstedt , Risk Management in Post-Trust Societies (London, Palgrave Macmillan 2005 ).

6

S Chakraborty , “Part I: The Role of Trust in Patient Non-Compliance: A Qualitative Case Study of Users of Statins for the Chronic Treatment of High-Cholesterol in New York City” (2013) 16 Journal for Risk Research 97 .

7

S Chakraborty , “Part II: The Role of Trust in Patient Non-Compliance: A Quantitative Case Study of Users of Statins for the Chronic Treatment of High-Cholesterol in New York City” (2013) 16 Journal for Risk Research 113 .

8

S Dohle , C Keller and M Siegrist , “Examining the Relationship between Affect and Implicit Associations: Implications for Risk Perception” (2010) 30 Risk Analysis 1116 2040904410.1111/j.1539-6924.2010.01404.x.

9

M Siegrist , C Keller and HAL Kiers , “A New Look at the Psychometric Paradigm of Perception of Hazards” (2005) 25 Risk Analysis 211 1578777010.1111/j.0272-4332.2005.00580.x.

10

S-H Oh , H-J Paek and T Hove , “Cognitive and Emotional Dimensions of Perceived Risk Characteristics, Genre-Specific Media Effects, and Risk Perceptions: The Case of H1N1 Influenza in South Korea” (2015) 25 Asian Journal of Communication 14 .

11

B Reynolds and M Seeger , “Crisis and Emergency Risk Communication as an Integrative Model” (2005) 10 Journal of Health Communication 43 1576444310.1080/10810730590904571.

12

JB Chung and GW Yun “Media and Social Amplification of Risk: BSE and H1N1 Cases in South Korea” (2013) 22 Disaster Prevention & Management 148 .

13

HJ Oh , T Hove , H-J Paek , BK Lee , H Lee and S Song , “Attention Cycles and the H1N1 Pandemic: A Cross-National Study of U.S. and Korean Newspaper Coverage” (2012) 22 Asian Journal of Communication 214 .

14

RE Kasperson et al, “The Social Amplification of Risk: A Conceptual Framework” (1988) 8 Risk Analysis 177 10.1111/risa.13926PMC1036013835861634.

15

TA Morton and JM Duck , “Communication and Health Beliefs: Mass and Interpersonal Influences on Perceptions of Risk to Self and Others” (2001) 28 Communication Research 602 .

16

C-L Coleman , “The Influence of Mass Media and Interpersonal Communication on Societal and Personal Risk Judgments” (1993) 20 Communication Research 611 .

17

BJ Holmes , “Communicating about Emerging Infectious Disease: The Importance of Research” (2008) 10 Health, Risk & Society 349 .

18 Morton and Duck, supra, note 15.

19

C Fife-Schaw and G Rowe , “Public Perceptions of Everyday Food Hazards: A Psychometric Study” (1996) 16 Risk Analysis 487 881934110.1111/j.1539-6924.1996.tb01095.x.

20 Oh et al, supra, note 10.

21 Slovic, supra, note 2.

22 SH Lim, “Again, *Sing-Jong* Flu?” *The Hankook Ilbo*, 6 January 2013 <http://news.hankooki.com/lpage/culture/201301/h2013010621111586330.htm>.

23 National Academies of Sciences, Engineering, and Medicine; Health and Medicine Division. National Academies Press (USA). Board on Global Health; Forum on Microbial Threats. Washington, DC.

24

B Fischhoff et al, “Public Understanding of Ebola Risks: Mastering an Unfamiliar Threat” (2017) 38 Risk Analysis 71 2859748010.1111/risa.12794.

25 US CDC, Crisis Communication Lifecycle <https://emergency.cdc.gov/cerc/cerccorner/article_051316.asp>.

26

SK Brooks et al, “The Psychological Impact of Quarantine and How to Reduce It: Rapid Review of the Evidence” (2020) 395 The Lancet 14 10.1016/S0140-6736(20)30460-8PMC715894232112714.

